# Engineering Foot-and-Mouth Disease Viruses with Improved Growth Properties for Vaccine Development

**DOI:** 10.1371/journal.pone.0055228

**Published:** 2013-01-25

**Authors:** Haixue Zheng, Jianhong Guo, Ye Jin, Fan Yang, Jijun He, Lv Lv, Kesan Zhang, Qiong Wu, Xiangtao Liu, Xuepeng Cai

**Affiliations:** State Key Laboratory of Veterinary Etiological Biology, National Foot and Mouth Diseases Reference Laboratory, Key Laboratory of Animal Virology of Ministry of Agriculture, Lanzhou Veterinary Research Institute, Chinese Academy of Agricultural Sciences, Lanzhou, China; Kantonal Hospital St. Gallen, Switzerland

## Abstract

**Background:**

No licensed vaccine is currently available against serotype A foot-and-mouth disease (FMD) in China, despite the isolation of A/WH/CHA/09 in 2009, partly because this strain does not replicate well in baby hamster kidney (BHK) cells.

**Methodology/Principal Findings:**

A novel plasmid-based reverse genetics system was used to construct a chimeric strain by replacing the P1 gene in the vaccine strain O/CHA/99 with that from the epidemic stain A/WH/CHA/09. The chimeric virus displayed growth kinetics similar to those of O/CHA/99 and was selected for use as a candidate vaccine strain after 12 passages in BHK cells. Cattle were vaccinated with the inactivated vaccine and humoral immune responses were induced in most of the animals on day 7. A challenge infection with A/WH/CHA/09 on day 28 indicated that the group given a 4-µg dose was fully protected and neither developed viremia nor seroconverted to a 3ABC antigen.

**Conclusions/Significance:**

Our data demonstrate that the chimeric virus not only propagates well in BHK cells and has excellent antigenic matching against serotype A FMD, but is also a potential marker vaccine to distinguish infection from vaccination. These results suggest that reverse genetics technology is a useful tool for engineering vaccines for the prevention and control of FMD.

## Introduction

Foot-and-mouth disease (FMD) is a highly infectious and economically important disease of ruminants. There are seven distinct serotypes: A, O, C, Asia 1 and South African Territories 1–3. These multiple subtypes reflect significant genetic variability [1], [2], [3], [4], [5]. FMD virus (FMDV) serotype A is one of the most antigenically divergent subtypes and is difficult to control by vaccination [6], [7]. There is great genetic and antigenic diversity among the strains of serotype A and often no cross-protection between them [8], [9], [10], [11], [12], [13], [14]. This is a result of the independent evolution of these viruses in different geographic regions, especially in Southeast Asia. In China, FMDV serotype A was first reported in Wuhan in January 2009, and was subsequently found in nine other areas in the Chinese mainland. Molecular epidemiological studies of the vp1 gene have shown that these isolates belong to the Asia topotype, which has caused endemic outbreaks in Southeast Asian countries in recent years [15], [16]. These strains continue to evolve and pose a serious threat to the livestock industry worldwide, and to previously FMD-free regions [17], such as South Korea, where this type of the virus was first reported in January 2010.

The currently available inactivated vaccines are often unable to control FMD caused by isolates from these countries, which highlights the need for custom-made vaccines for use in specific geographic regions [16]. However, the development of a new seed virus for the production of a potent vaccine is both time-consuming and expensive. The 50% tissue culture infective dose (TCID_50_) and the 50% lethal dose (LD_50_) in mice of field isolates are usually lower than those of the seed viruses of established vaccine strains. Even serial passages often do not improve these results, and low antigen yields are produced. Furthermore, some antigens derived from epidemiologically relevant field isolates are unstable or induce only a narrow immune response. Therefore, many field isolates are unsuitable as starting materials for FMD vaccines and producers have to test high numbers of field isolates by serial passaging and preparing trial vaccines. It is often preferred to use an established vaccine strain that induces a broad immune response, but this approach clearly has limitations.

To circumvent some of the problems of vaccine strain selection and adaptation, this study investigated an alternative procedure. It has been proposed that the antigenic determinants in an infectious genome-length cDNA of a vaccine strain can be replaced with those of appropriate field strains, producing custom-made FMDV chimeras for use in vaccine production [18], [19]. We constructed a chimera by replacing the principal antigenic P1 gene in an existing cDNA clone of vaccine strain O/CHA/99 with the P1 gene from the field strain, A/WH/CHA/09. The chimeric virus exhibited comparable growth characteristics in culture and infection kinetics to the parental O/CHA/99 strain and the field A/WH/CHA/09 field strain, which suggests that the chimera is a promising vaccine candidate.

## Results

### Construction of prA/P1-FMDV

**Figure 1:**
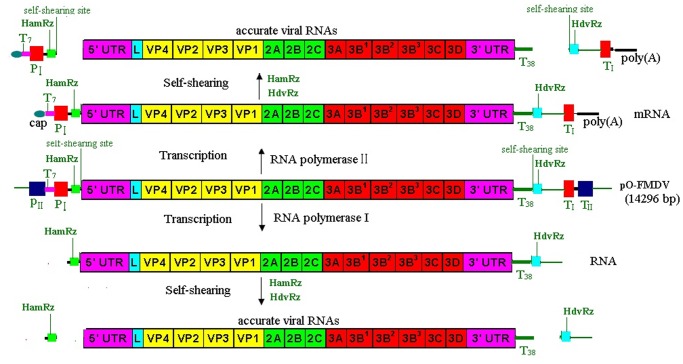
The pol I/pol II unidirectional transcription system. The pO-FMDV plasmid contains the RNA polymerase II promoter (pIICMV) of the human cytomegalovirus and the polyadenylation signal (aIIBGH) of the gene encoding bovine growth hormone. Inserted between these elements is a murine terminator (TI) and the sequence of the mouse RNA polymerase I promoter (PI). The full-length cDNA of the genome of type Asia1 FMDV JS/CHA/05 strain, flanked by hammerhead ribozyme (HamRz) and hepatitis delta ribozyme (HdvRz) sequences, was inserted between the P_I_ and T_I_, resulted in the recombinant plasmid pO-FMDV containing a polymerase I and II transcription cassette, which permits intracellular transcription of the viral RNA (vRNA) with HamRz and HdvRz. Transcription by RNA polymerase II is expected to result in an mRNA with a 5′ cap structure and a 3′ poly A tail (up), transcription by RNA polymerase I is expected to result in a (+)RNA without a 5′ cap structure and a 3′ poly A tail (low). After the self-cleaving of HamRz and HdvRz, the synthesized positive-strand RNAs would generate into the viral full-length viral RNA transcripts with authentic 5′ and 3′ ends and have infective in vivo. HamRz: Hammerhead ribozyme; HdvRz: Hepatitis delta virus ribozyme; P_II_: polymerase II promoter; P_I_ polymerase I promoter; P_II_: polymerase II promoter; T_II_: polymerase II terminator; T_I_: polymerase I terminator; T38∶3′ poly(T) tail with 38 Ts. A38∶3′ poly(A) tail with 38 As.

A full-length cDNA clone of the rA/P1-FMDV strain was assembled using a construction strategy, which replaced the P1 gene in the O/CHA/99 vaccine strain with that from the A/WH/CHA/09 epidemic strain (see Materials and Methods for details). One cDNA clone, designated prA/P1-FMDV, was produced. There were only two amino acid differences between the two P1 proteins from the rA/P1-FMDV and A/WH/CHA/09 strains at positions 208 and 211 of antigenic site 1 (V_208_KQT_211_L in rA/P1-FMDV, A_208_KQL_211_L in A/WH/CHA/09). The full-length cDNA was flanked by the hammerhead ribozyme (HamRz) and the hepatitis delta ribozyme (HdvRz) sequences. These were arranged downstream of the two promoters (cytomegalovirus [CMV] and pol I promoter) and upstream of the terminators and the polyadenylation signal, as described in Figure 1 (see Materials and Methods for details).

### Characterization of the Recovered Virus

**Figure 2:**
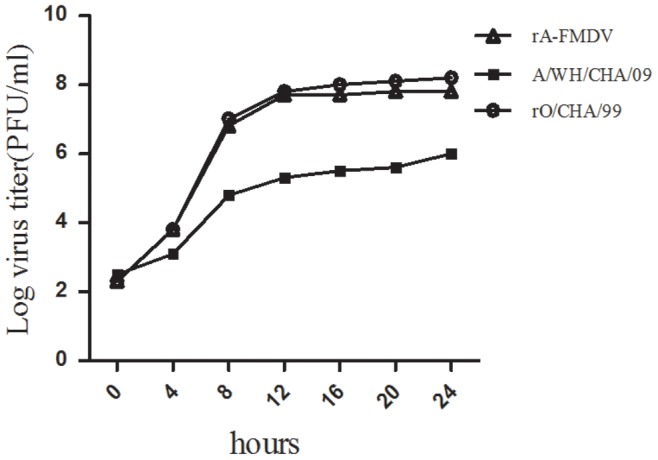
One-step growth curves of the recombinant viruses, rA/P1-FMDV and rO/CHA/99, and the epidemic virulent strain, A/WH/CHA/09, as determined in BHK-21 cells.

In order to define the differences among the rA/P1-FMDV, A/WH/CHA/09 and O/CHA/99 strains more clearly, their growth characteristics were compared by one-step growth kinetics assays. Although rA/P1-FMDV showed similar growth properties to O/CHA/99, which were consistent with their higher titer, the A/WH/CHA/09 virus grew much more poorly in BHK cells, which was consistent with its smaller plaque size and lower titer (Figure 2).

**Figure 3:**
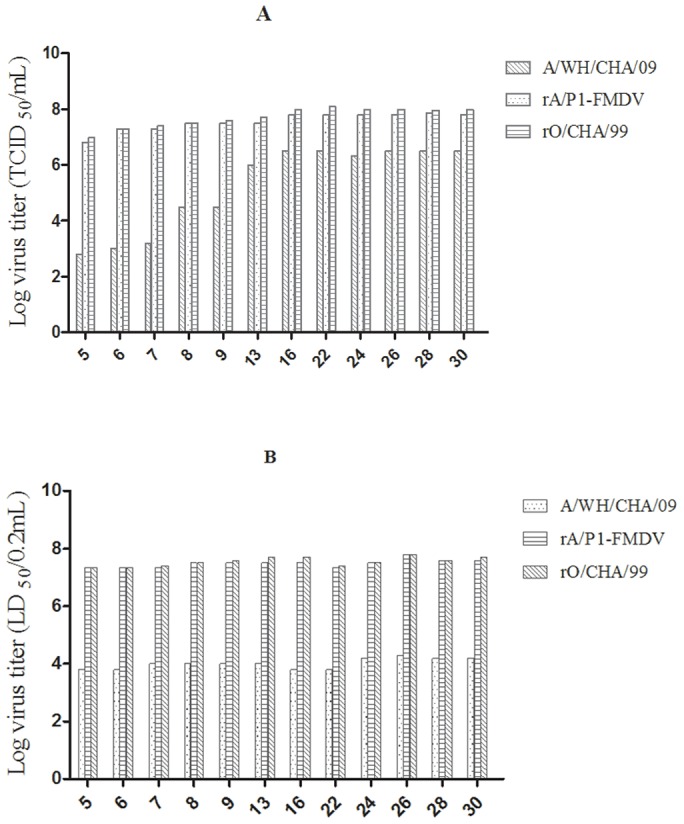
The 50% tissue culture infection dose (TCID_50_) and 50% lethal dose (LD_50_) of the recombinant viruses, rA/P1-FMDV and rO/CHA/99, and the epidemic virulent strain, A/WH/CHA/09. A: TCID50, B: LD50.

To compare and characterize the properties of the virus further between passages 5 to 30, the titers (TCID_50_) of the viruses achieved after overnight growth were determined in BHK-21 cell lines. The LD_50_ was determined in mice using the Reed-Muench formula [20] (Figure 3). The titers of rA/P1-FMDV were higher than those of A/WH/CHA/09 (Figure 3). The ID_50_ in cattle was 6.0 for rA/P1-FMDV, which was similar to the value for A/WH/CHA/09. rA/P1-FMDV viruses between passages 5 to 30 were sequenced and compared, and the results showed that there was a high level of homology with hardly any amino acid changes between them (Table 1).

**Table 1:** Mutant nucleotide and amino acid changes of the recombinant virus, rA/P1-FMDV, obtained in BHK–21 cells at passages 5, 13, 22, 26 and 30.

Plasmid nameor passage time	Mutant nucleotide and amino acid changes related to prA/P1-FMDV^a^											
	5′UTR^b^			L	VP2	VP3	VP3	VP1	2C	3A	3A	3D
	261^c^	701	995	1238	2230	2637	2827	3628	5256	5517	5694	7056
prA/P1-FMDV	T	A	T	A(Thr^d^)	A(Lys)	A(Pro)	A(Glu)	C(His)	T(Val)	A(Gln)	C(Asp)	C(Leu)
5^e^	T	A	T	A(Ser)	A(Lys)	A(Pro)	A(Glu)	A(Gln)	C(Val)	A(Gln)	T(Asp)	T(Leu)
13	G	A	C	A(Ser)	G(Lys)	G(Pro)	G(Glu)	A(Gln)	T(Val)	A(Gln)	T(Asp)	T(Leu)
22	G	T	C	A(Ser)	A(Lys)	G(Pro)	A(Glu)	A(Gln)	C(Val)	A(Gln)	T(Asp)	T(Leu)
26	G	A	C	A(Ser)	A(Lys)	G(Pro)	A(Glu)	A(Gln)	C(Val)	A(Gln)	T(Asp)	T(Leu)
30	G	A	C	A(Ser)	A(Lys)	G(Pro)	A(Glu)	A(Gln)	C(Val)	G(Gln)	T(Asp)	T(Leu)

a Full-length genome sequencing of five viruses was performed and compared to plasmid prA/P1-FMDV.

b Virus region.

e Nucleotide position.

d Corresponds to amino acid or amino acid substitution.

e Passage time.

### Anti-FMDV Neutralizing Antibody in Cattle

Neutralizing antibody titers were measured at 0, 7, 14, 21 and 28 days post-vaccination (dpv) following the virus neutralization test (VNT) method. All cattle vaccinated with rA/P1-FMDV antigens developed detectable FMDV neutralizing antibody responses at 7 dpv, and high levels were reached at 14 dpv. At 21 and 28 dpv, the neutralizing antibody level was maintained at the same level or higher; in number 1264, it reached a titer of 2.4. In contrast, neutralizing antibody levels in the two unvaccinated controls were not boosted (Table 2).

**Table 2:** Immune response in cattle after vaccination.

Animal No	Vaccine^a^ dose (µg)	Neutralizing antibody titre^b^					
		Post-vaccination (d)^c^					
		0 d	7d	14d	21d	28d	
**1251**	**2**	**<0.6**	**0.6**	**0.6**	**0.9**	**1.0**	
1252	2	**<**0.6	1.3	1.0	1.3	1.7	
1256	2	**<**0.6	0.9	0.9	0.9	0.9	
1262	2	**<**0.6	1.7	2.0	2.0	2.0	
**1266**	**2**	**<0.6**	**0.9**	**0.9**	**1.0**	**1.0**	
1269	2	**<**0.6	1.3	1.7	1.3	1.2	
1274	2	**<**0.6	1.0	1.0	1.0	1.3	
1287	2	**<**0.6	1.0	1.7	1.7	1.7	
1255	4	**<**0.6	2.0	2.0	2.1	2.3	
1259	4	**<**0.6	2.0	2.0	2.0	2.0	
1264	4	**<**0.6	2.0	2.0	2.3	2.4	
1277	4	**<**0.6	1.7	1.7	1.7	1.5	
1278	4	**<**0.6	1.3	1.7	1.7	1.7	
1279	4	**<**0.6	1.0	1.7	2.0	2.0	
1282	4	**<**0.6	1.3	1.5	2.0	2.0	
1286	4	**<**0.6	1.0	1.5	1.7	2.0	
**1265**	**0**	**<0.6**	**<0.6**	**<0.6**	**<0.6**	**<0.6**	
**1280**	**0**	**<0.6**	**<0.6**	**<0.6**	**<0.6**	**<0.6**	

a Bovines were vaccinated with vaccine prepared from rA/P1-FMDV and challenged 28 days later.

b FMDV-specific antibody titer reported as the serum dilution by VNT method. The antibody titers were calculated as the log_10_ of the reciprocal of the final serum dilution that neutralized 100 TCID_50_ of virus in 50% of the wells.

c Days post-vaccination.

### Immunogenicity of Chimeric Virus

Vaccine matching relationship (*r*) values for rA/P1-FMDV, A/WH/CHA/09 and O/CHA/99 against neutralizing rA/P1-FMDV antibody are summarized in Table 3. As expected, the highest *r* values were obtained from the cattle vaccinated with the chimera, in which antibodies were cross-reactive with rA/P1-FMDV and A/WH/CHA/09. The *r* values for O_/_CHA/99 were lower.

**Table 3:** Vaccine matching ‘r’ values of rA/P1-FMDV, A/WH/CHA/09 and O_/_CHA/99.

Antisera^a^	Vaccine dose (µg)	‘r’ values against a selection of viruses^b^		
		rA/P1-FMDV	A/WH/CHA/09	O_/_CHA/99
1255	4	**1.00**	**0.95**	0.00
1259	4	**1.00**	**0.95**	0.10
1264	4	**1.00**	**0.96**	0.00

a BEI-inactivated virus rA/P1-FMDV, 4 µg/dose.

b by VNT.

* Figures shown in bold are indicative of likely protection by the relevant vaccine strain. In the case of VNT: r1≥0.3 suggests that there is a close relationship between field isolate and vaccine strain. A potent vaccine containing the vaccine strain is likely to confer protection. r1≤0.3 suggests that the field isolate is so different from the vaccine strain that the vaccine is unlikely to protect.

### Protective Efficacy in Cattle

The cattle were monitored daily for clinical signs of disease up to 10 days post-challenge. The progression of the disease was evaluated using a previously described scoring system with slight modifications [21]. Individual clinical signs were scored as follows: lameness, 1; lesions on one foot, 1; lesions on two feet, 2; lesions on three feet, 3; lesions on four feet, 4; lesions on the tongue, mouth or gingiva, 1. The scores were then calculated. A cow with lesions on any foot other than that in which the injection was made was also regarded as lame. Cattle could therefore score a maximum of six points. When the disease was judged to be severe, the animal was killed by injection of an overdose of pentobarbitone (Euthatal; Merial Animal Health, Lyon, France) followed by exsanguination.

The group of cattle that received a full vaccine dose was fully protected against needle challenge, as determined by the absence of both injection site and generalized lesions and pyrexia (their temperatures remained below 39.5°C). The group that received a half dose was 75% (6/8) protected against needle challenge. This was in contrast to the unvaccinated controls, which developed pyrexia, lesions on the muzzle and severe lesions on all four feet within 10 d of needle challenge (Table 4).

**Table 4:** The clinical signs and protection against challenge with 10 000 BID_50_ of A/WH/CHA/09.

Animal No.	Vaccine dose (µg)	1 dpc	2 dpc	3 dpc	4 dpc	5 dpc	6 dpc	7 dpc	8 dpc	9 dpc	10 dpc	Viraemia	Protection
1251	**2**	0	1	1	1	1	2	2	0	0	0	Yes	No
1252	2	0	1	0	0	0	0	0	0	0	0	No	Yes
1256	2	0	1	0	0	0	0	0	0	0	0	No	Yes
1262	2	0	0	0	0	0	0	0	0	0	0	No	Yes
1266	**2**	0	1	1	1	1	1	0	0	0	0	Yes	No
1269	2	0	0	0	0	0	0	0	0	0	0	No	Yes
1274	2	0	0	0	0	0	0	0	0	0	0	No	Yes
1287	2	0	0	0	0	0	0	0	0	0	0	No	Yes
1255	4	0	0	0	0	0	0	0	0	0	0	No	Yes
1259	4	0	0	0	0	0	0	0	0	0	0	No	Yes
1264	4	0	0	0	0	0	0	0	0	0	0	No	Yes
1277	4	0	0	0	0	0	0	0	0	0	0	No	Yes
1278	4	0	1	0	0	0	0	0	0	0	0	No	Yes
1279	4	0	0	0	0	0	0	0	0	0	0	No	Yes
1282	4	0	0	0	0	0	0	0	0	0	0	No	Yes
1286	4	0	0	0	0	0	0	0	0	0	0	No	Yes
1265	**0**	0	1	1	1	1	1	4	4	4	4	Yes	No
1280	**0**	0	1	1	1	5	5	5	5	5	5	Yes	No

dpc indicates day post-challenge.

### Detection of Subclinical Infection

#### Detection of antibodies against NSP 3ABC

As expected, antibodies against the FMDV nonstructural protein (NSP) 3ABC were detected in both unvaccinated control animals from day 7 post-challenge until the last day of sampling (Table 6). Animals 1251, 1252, 1266, 1265 and 1280 were consistently seropositive between days 14 and 32. All of the other vaccinated cattle remained NSP seronegative (Table 5).

**Table 5:** NSP serology was detected by the NSP-3ABC detection kit.

Animal number	0 dpc	7 dpc	14 dpc	21 dpc	28 dpc	32 dpc
1251	**−**	**−**	+	+	+	+
1252	**−**	**−**	**−**	**−**	**−**	**−**
1256	**−**	**−**	**−**	**−**	**−**	**−**
1262	**−**	**−**	**−**	**−**	**−**	**−**
1266	**−**	**−**	**+**	**+**	**+**	**+**
1269	**−**	**−**	**−**	**−**	**−**	**−**
1274	**−**	**−**	**−**	**−**	**−**	**−**
1287	**−**	**−**	**−**	**−**	**−**	**−**
1255	**−**	**−**	**−**	**−**	**−**	**−**
1259	**−**	**−**	**−**	**−**	**−**	**−**
1264	**−**	**−**	**−**	**−**	**−**	**−**
1277	**−**	**−**	**−**	**−**	**−**	**−**
1278	**−**	**−**	**−**	**−**	**−**	**−**
1279	**−**	**−**	**−**	**−**	**−**	**−**
1282	**−**	**−**	**−**	**−**	**−**	**−**
1286	**−**	**−**	**−**	**−**	**−**	**−**
1265	**−**	**+**	**+**	**+**	**+**	**+**
1280	**−**	**+**	**+**	**+**	**+**	**+**

dpc indicates day post-challenge.

+ indicates positive samples, **−** indicates positive samples.

**Table 6:** Quantitative rRT-PCR results for blood, nasal and oropharyngeal samples taken between days 0 and 32 post-challenge.

Animal number	blood	nasal	oropharyngeal
1251	**3 (33.4) 5 (35.2)**	**7 (35.8)**	**7 (23.6)**
1252	**−**	**−**	**−**
1256	**−**	**−**	**−**
1262	**−**	**−**	**−**
1266	**3 (34.41)**	**7 (35.5)**	**7 (25.5)**
1269	**−**	**−**	**−**
1274	**−**	**−**	**−**
1287	**−**	**−**	**−**
1255	**−**	**−**	**−**
1259	**−**	**−**	**−**
1264	**−**	**−**	**−**
1277	**−**	**−**	**−**
1278	**−**	**−**	**−**
1279	**−**	**−**	**−**
1282	**−**	**−**	**−**
1286	**−**	**−**	**−**
1265	**2 (32.4)**	**2 (30.4) 5 (31.2)**	**7 (23.5) 14 (30.1) 28 (31.0) 32 (33.0)**
1280	**2 (36.5)**	**2 (33.14) 5 (35.0)**	**7 (24.1)**

+ indicates positive samples.

Numbers indicate the day of the positive sample; brackets indicate the ct-value (ct-values less than 35 were considered positive).

#### Detection of viral RNA by rRT-PCR

FMDV RNA was detected in oropharyngeal samples from three chimera-vaccinated animals (1251, 1252 and 1266) on day 7 post-challenge. FMDV RNA was also detected in nasal and oropharyngeal samples taken from the unvaccinated controls on days 2 and 5 post-challenge (Table 6).

## Discussion

Vaccination plays a major role in the prevention of FMD and its complications. However, the constant antigenic drift and periodic antigenic shift require that FMD vaccines should be updated frequently to be effective against circulating strains. Currently licensed FMDV vaccines are produced in BHK cells. Occasionally, an epidemic virulent strain, such as A/WH/CHA/09, does not replicate well in BHK cells and is thus considered to be unsuitable as a candidate vaccine.

FMDV replication has been found to depend on stem-loop (SL) structures in the 3′ noncoding region of the viral RNA [22]. The deletion of SL2 inhibits viral infectivity in vitro, and the deletion of SL1 downregulates viral replication in association with impaired negative-strand RNA synthesis, leading to viruses with slower growth kinetics [22]. In our previous study, the analysis of the complete genome of A/WH/CHA/09 showed that the 3′ noncoding region folds into three SL structures: SL1 is split into two SLs, whereas SL2 retains its primary structure. To investigate the role of mutations of SL structures in the replication of FMDV, several related recombinant strains were produced by introducing SL structural mutations into the O/CHA/99 strain. Mutations of SL1 were found to be responsible for viruses that grew slowly in BHK cells (results not published). We therefore selected the infectious full-length cDNA of O/CHA/09 without SL structural mutations as the backbone for the construction of prA/P1-FMDV. The virus derived from prA/P1-FMDV grew as well in culture as O/CHA/99 and had a shortened time to CPE; furthermore, the rA/P1-FMDV titer was markedly increased after 12 passages in BHK cells compared with its parental A/WH/CHA/09 strain. rA/P1-FMDV viruses between passages 5 to 30 were sequenced and compared, and were shown to have a high level of homology with hardly any amino acid differences (Table 1), which demonstrated that these viruses have stable genetic characteristics. The ID_50_ of rA/P1-FMDV in cattle was similar to that of A/WH/CHA/09.

The recombinant vaccine candidate, rA/P1-FMDV, not only exhibits the excellent growth of O/CHA/99, but is also a good antigenic match with A/WH/CHA/09. Vaccine matching showed that sera from cattle vaccinated with the chimera were cross-reactive with rA/P1-FMDV and A/WH/CHA/09, which indicates that rA/P1-FMDV is likely to confer protection. Cattle are economically important, and cloven-hoofed animals can be used as a model for FMDV infection; thus, we designed an experiment to verify whether rA/P1-FMDV might be used to prepare an FMD vaccine. We followed the bovine potency test protocol described by the OIE to determine the potency of the recombinant vaccine. Eight cattle (group one) were vaccinated intramuscularly with a half dose (1 ml) of vaccine and another eight cattle (group two) with a full dose (2 ml). By 2 weeks post-vaccination, antibodies in the vaccinated cattle had reached a high titer. This level was maintained thereafter, whereas the titer in the control group remained low. After being challenged with the field isolate A/WH/CHA/99, the eight cattle that had received a full vaccine dose were fully protected against needle challenge. Those that had received a half dose were 75% (6/8) protected; in two animals (1251 and 1266), the disease was delayed and the clinical symptoms were less severe. The two unvaccinated cattle developed lesions on all feet and inside their mouths on the second day. A cell-mediated immune response was probably involved in this protection [23], which would explain why animal 1256 had a neutralizing antibody titer of 0.9, but was still protected.

To determine whether the recombinant strain rA/P1-FMDV could be used to produce an effective vaccine, samples from both vaccinated and unvaccinated animals were analyzed by rRT-PCR and by ELISA for NSP 3ABC. The rRT-PCR results were consistent with those of the ELISA. Two vaccinated animals (1251 and 1266) had subclinical symptoms. This low level of subclinical disease indicates the efficacy of the chimeric vaccine, which may be as effective as high-potency vaccines that have already been reported to inhibit local viral replication and prevent the carrier state [24], [25], [26], [27], [28], [29]. The evidence supports the use of rA/P1-FMDV as an emergency vaccine. The chimera contains a recombinant antigenic site 1; at positions 208–212, the amino acid sequence is VKQTL in rA/P1-FMDV and AKQLL in A/WH/CHA/09. This difference might induce different monoclonal antibodies in host animals, which could be used as a marker to distinguish infection from vaccination. Thus, the chimera rA/P1-FMDV is also a potential marker vaccine. Further studies are required to determine whether the changes reported here affect the immune response.

We selected the A/WH/CHA/09 strain for the production of a custom-made vaccine because of its great genetic and antigenic diversity; furthermore, there is often no cross-protection among the members of serotype A as a result of the independent evolution of these viruses in different geographic regions, especially in Southeast Asian countries [8], [9], [10], [11], [12], [13], [14]. The prevention and control by vaccination is difficult, which makes the argument for the development of custom-made vaccines for use in these countries [16]. China was serotype A FMD-free for over 40 years until epidemic strains of the Asia topotype appeared in 2009. There are currently no vaccine strains that match well with epidemic strains in this region, and the epidemic strain A/WH/CHA/09 is not suitable for vaccine production because of its slow growth. We therefore utilized a novel plasmid-based reverse genetics technique to develop a custom-made vaccine that could be used to respond to a pandemic alert. The novel RNA polymerase I and II driven plasmid-based reverse-genetics system was developed containing a polymerase I and II transcription cassette, which permits the intracellular transcription of viral RNA with HamRz and HdvRz sequences. After the self-digestion of the HamRz and HdvRz ribozymes, full-length viral genomic RNA transcripts are generated with accurate 5′ and 3′ ends. Therefore, the plasmid has in vivo infectivity and is therefore similar to a “DNA virus”. In this study, through the direct transfection of the plasmid into the BHK-21 cell line and its inoculation into a host animal, FMDV was efficiently and stably rescued from the plasmid. Thus, this reverse-genetics system will be useful for future basic mechanistic research and vaccine investigations. It will be particularly useful in rescuing viruses for which there are currently no suitable cell culture systems. Our current study showed that the two promoters (CMV and pol I) permit the intracellular transcription of viral RNA, which improve genome expression and increase the rescue efficiency compared with ones that contain either the pol I or the pol II promoter (results not published). Reverse genetics is a powerful tool in the design of FMD vaccines, not only for improving growth, but also for improving antigenic matching. This strategy has successfully addressed the problems described above and provided a potential marker vaccine. More importantly, it can be extended to other antigenically diverse FMDV types in other regions or countries.

## Materials and Methods

### Ethics Statement

All animal work was approved and conducted according to the requirements of the Gansu Animal Experiments Inspectorate and the Gansu Ethical Review Committee (License no. SYXK [GAN] 2010-003).

### Cell Lines and Viruses

BHK cells, strain 21 (maintained in basal medium Eagle [Invitrogen, Carlsbad, CA] containing 7% fetal calf serum [Delta Bioproducts, Atascadero, CA] and 10% tryptose phosphate broth) were used for transfection and for TCID_50_, LD_50_ in mice and vaccine matching tests [30], [31].

O/CHA/99 that originated from Chinese Hong Kong in 1999, the master donor virus for FMD vaccine, was previously used to construct an infectious genome-length backbone in our laboratory [32]. Based on this backbone, a novel RNA polymerase I and II driven plasmid-based reverse-genetics system (pO-FMDV) has been developed containing a polymerase I and II transcription cassette that permits intracellular transcription of viral RNA with the hammerhead ribozyme (HamRz) and the hepatitis delta ribozyme (HdvRz) sequences. After the self-digestion of the HamRz and HdvRz ribozymes, the full-length viral genomic RNA transcripts with true 5′ and 3′ ends were generated (Figure 1). In the reverse genetics system, the full-length viral cDNA is flanked by the HamRz and HdvRz sequences, which are arranged downstream of the two promoters (CMV and Pol I promoter), and upstream of the terminators and the polyadenylation signal. Therefore, the plasmid was similar to a “DNA virus”. Through the direct transfection of the plasmid into the BHK-21 cell line and its inoculation into a host animal, FMDV was efficiently rescued from the plasmid.

A/WH/CHA/09 is an epidemic virulent strain that originates from Wuhan, China in 2009. Its P1 gene was selected for exchange with the corresponding region of the O/CHA/99 strain.

### RNA Extraction and cDNA Synthesis

RNA was extracted from a tissue culture sample of A/WH/CHA/09 using RNeasy (Qiagen, Hilden, Germany) according to the manufacturer’s specifications and used as a template for cDNA synthesis. SuperScript Moloney murine leukemia virus reverse transcriptase (Life Technologies, Carlsbad, CA) and random hexamers as primers were used for the reverse transcription (RT) reactions, which were conducted for 1 h at 42°C.

### Construction of Recombinant Genome-length cDNAs and Sequencing

The RT production was used as a template, the P1 cDNA of A/WH/CHA/09 was obtained by PCR amplification (using sense primer AP1-F [5′-TTTTC CTTAAG GGA CAG GAA CAT GCT GTG TTT GCC TGC GT-3′; the sequence of the restriction endonuclease *Afl*II site is underlined] and antisense primer AP1-R [5′-TATTTT CAC CGG TG CAA TAA TTT TCT GCT TGT GTC TGT C-3′; the sequence of the restriction endonuclease *SgrA*I site is underlined]) using AdvanTaq DNA Polymerase (Clontech, Mountain View, CA).

The PCR amplification thermal program to produce single-stranded PCR product consisted of an initial denaturation step at 94°C for 2 min, followed by 35 cycles of 94°C for 30 s, 57°C for 30 s, and 72°C for 3 min. This was followed by a final extension of 72°C for 10 min. Then, the PCR product was purified with the use of the Wizard SV Gel and PCR Clean-Up System Kit (Promega, Shanghai, China) and treated with the restriction endonucleases, *Afl*II and *SgrA*I (New England Biolabs, Massachusetts, USA). Meanwhile, the pO-FMDV plasmid was digested with the same restriction endonucleases. The treated PCR fragment was then inserted directly into the treated pO-FMDV backbone, and the resulting plasmid was designated as prA/P1-FMDV.

The nucleotide sequence of prA/P1-FMDV was determined using selected oligonucleotides and an ABI PRISM BigDye Terminator v3.0 Ready Reaction Cycle Sequencing Kit (Applied Biosystems, Foster City, CA), followed by resolution on an ABI PRISM 310 Genetic Analyzer (Applied Biosystems). To confirm the successful insertion into pO-FMDV, the two cloning sites (*Afl*II and *SgrA*I) of the intermediate A/O constructs were also verified through nucleotide sequencing.

### Transfection of BHK-21 Cells with prA/P1-FMDV

The purified plasmid prA/P1-FMDV and pO-FMDV were prepared using QIAGEN Plasmid Midi Kits (Qiagen, Hilden, Germany) according to the manufacturer’s protocol. The plasmid was transfected directly into subconfluent BHK-21 cells in 10 ml plates using Lipofectamine™ 2000 (Invitrogen, Carlsbad, CA) according to the manufacturer’s protocol. Six hundred microliters of Opti-MEM I reduced serum medium (Invitrogen) was added to the monolayer and the cells were incubated at 37°C for 1 h. The plasmid (10 µl plasmid at 2 µg/µl) was diluted in 590 µl Opti-MEM I. Simultaneously, 50 µl Lipofectamine™ 2000 was diluted in 550 µl Opti-MEM I and incubated for 5 min at room temperature. The diluted plasmid was combined with diluted Lipofectamine™ 2000 in a total volume of 1200 µl, mixed gently and incubated for 20 min at room temperature. The mixture containing plasmid was added to the BHK-21 cell monolayer. After 5–6 h of incubation at 37°C, the cells were washed three times with Hank’s buffer and maintained in Dulbecco’s modified Eagle’s medium supplemented with 10% fetal bovine serum for a further 48 h at 37°C in a humidified 5% CO_2_ incubator. The plasmid pcDNA3.1(+), as a negative control, was also used to transfect BHK-21 cells by the procedure as described above. After 48 h, the supernatants were harvested. To increase the recovered virus titers, after freezing and thawing three times, the recovered viruses (rA/P1-FMDV), which were harvested from the supernatants by centrifugation at 5,000×*g* for 10 min at 4°C, were passaged 25 times in BHK-21 cells.

### Growth Characterization of rA/P1-FMDV, A/WH/CHA/09 and rO/CHA/99

The rescued virus, rA/P1-FMDV, rO/CHA/99 and the epidemic strain, A/WH/CHA/09, were harvested in BHK-21 cells at the 5^th^ and 30^th^ passages. A 10-fold dilution of the rescued virus was prepared in PBS containing 1% FBS, and groups of 3-day-old mice (n  = 5 each group) were inoculated intraperitoneally with 0.2 ml of virus diluted in PBS. The mice were observed for 72 h after infection and the 50% lethal dose (LD_50_) was determined according to the method described by Reed and Muench [20].

The 50% tissue culture infective dose (TCID_50_) was also determined on confluent BHK-21 cell cultures in 96-well plates. Subconfluent monolayers were infected with serial 10-fold dilutions of a rescued virus suspension. After incubation for 1 h at 37°C, the cells were washed with medium and cultured in DMEM with 2% FBS under the same conditions. Supernatants were sampled from the infected cell cultures at various time points in order to calculate the virus titers, which were expressed as the 50% tissue culture infective dose (TCID_50_) per milliliter.

The growth kinetics of the viruses were determined in BHK-21 cells by one-step growth kinetics assays. Confluent monolayers in 60-mm diameter plates were infected at a multiplicity of infection (MOI) of 10 PFU per cell with rA/P1-FMDV, O/CHA/99 and A/WH/CHA/09. After adsorption for 1 h, the monolayers were washed with 0.01 M phosphate-buffered saline (PBS; pH7.4), and maintained in DMEM supplemented with 2% FBS at 37°C with 5% CO_2_. The virus-infected supernatants were collected at 4, 8, 12, 16 and 24 h after inoculation. Virus titers (plaque-forming units (pfu) mL^−1^) were determined on BHK-21, as described elsewhere [33].

### Antigen and Vaccine Production

Clarified virus supernatants from BHK-21 cultures infected during the 12^th^ passage of the rA/P1-FMDV strain were used to inoculate roller bottle cultures of BHK-21 cells (1,500 cm^2^, 5 rollers). On the appearance of 100% CPE, the viruses were harvested, BEI-inactivated and purified by a sucrose density gradient. Then, 10% of the clarified cell culture supernatants were kept as a live virus and stored at −70°C for in vitro assays.

Two water-in-oil-in-water vaccines were prepared from rA/P1-FMDV and A/WH/CHA/09, respectively, each containing 4 µg of BEI-inactivated, 30% (w/v) sucrose density gradient purified 146S FMDV antigen; Montanide ISA 206 (Seppic) was used as the oil adjuvant and mixed with the aqueous phase at a ratio of 50∶50. In both cases, the content of the sucrose-purified antigen was determined previously by evaluating the optical density of samples at 260 nm.

### Immunization of Cattle and Detection of Neutralizing Antibody by VNT

VNT was performed to determine neutralizing antibody titers for the screening of candidate cattle before vaccination. Cattle with a potency lower than 0.6 were housed in disease-secure isolation facilities at Lanzhou Veterinary Research Institute. Eighteen cattle of 6–7 months of age were housed separately in two groups of eight (vaccinated animals: group one animal numbers were 1251, 1252, 1256, 1262, 1266, 1269, 1274 and 1287, group two animal numbers were 1255, 1259, 1264, 1277, 1278, 1279, 1282 and 1286) and one group of two (unvaccinated controls: animal numbers were 1265 and 1280). The eight cattle in group one were vaccinated intramuscularly with a 1 ml (2 µg) dose and the eight cattle in group two with a 2 ml (4 µg) dose. The negative control cattle, 1265 and 1280, were used as unvaccinated controls. Sera were collected at 0, 7, 14, 21 and 28 dpv. Neutralizing antibodies against rA/P1-FMDV vaccine in serum samples collected from pigs were measured with a VNT at 0, 7, 14, 21 and 28 dpv, according to the method described in the Manual of Diagnostic Tests and Vaccines for Terrestrial Animals by Office International des Epizooties (2009) using IBRS-2 cells in microtiter plates. The antibody titers were calculated as the log_10_ of the reciprocal of the final serum dilution that neutralized 100 TCID_50_ of virus in 50% of the wells.

### Vaccine Matching

Vaccine matching was performed by comparing the reactivity of bovine antisera against rA/P1-FMDV, the field isolate A/WH/CHA/09 and O/CHA/99 using a virus neutralization test. An r value of less than 0.3 indicates that a vaccine of normal potency is unlikely to give adequate protection [30], [31].

### A/WH/CHA/09 Field Strain Challenge and 3ABC Antibody Detection by 3ABC-ELISA

According to the standard protocol of the OIE, all animals were challenged by intradermal inoculation at two sites in the tongue, with 10,000 BID_50_ of the field strain A/WH/CHA/09 at 28 dpv. Clinical signs were recorded daily. Clotted and heparinized blood was collected on days 0, 2, 5, 7, 14, 21, 28 and 32 post-challenge, along with oropharyngeal fluid using a probing and nasal swabs. In addition, serum samples were taken at intervals until 32 days post-challenge and examined for the presence of antibodies to the FMDV NSP 3ABC. The NSP 3ABC detection kit was prepared by Lanzhou Veterinary Research Institute [34]. Samples were considered positive if the percentage of inhibition was 50% or greater.

### Detection of Viral RNA by rRT-PCR

One-step rRT-PCR was performed on each sample according to the procedure described by Shaw *et al.*, 2007 [35]. The TaqMan® primers (SA-IR-219-246F, SA-IR-315-293R) and probe (SAmulti2-P-IR-292-269R) used in the assay, targeting conserved sequences in the internal ribosomal entry site within the 5′-untranslated region of the FMDV genome, have been reported previously [36]. The rRT-PCR master mix comprised 12.5 µl of 2× reaction mix (SuperScript III/Platinum Taq One-Step rRT-PCR Kit; Invitrogen, Carlsbad, USA), 2.0 µl of each primer (both at 10 pmol/µl), 1.5 µl probe (5 pmol/µl), 1.5 µl nuclease-free H_2_O (Promega, Madison, WI) and 0.5 µl High Fidelity Enzyme Mix (SuperScript III/Platinum Taq One-Step rRT-PCR Kit; Invitrogen) per reaction. Briefly, 20 µl of pre-prepared rRT-PCR master mix was added to the appropriate number of wells in a 96-well optical reaction plate (Stratagene, La Jolla, CA) followed by 5 µl of RNA. The reaction was performed in an Mx4000 Sequence Detection System (Stratagene) using the following thermal profile: 30 min at 60°C, one cycle; 10 min at 95°C, one cycle; 15 s at 95°C, 1.06 min at 60°C, 50 cycles. The results from all samples were analyzed using Stratagene® MxPro™ QPCR software and a CT value was assigned to each reaction as described previously [36]. Samples with a CT value of 35 or less were considered positive.
